# Clinical malaria in African pregnant women

**DOI:** 10.1186/1475-2875-7-27

**Published:** 2008-01-30

**Authors:** Azucena Bardají, Betuel Sigauque, Laia Bruni, Cleofé Romagosa, Sergi Sanz, Samuel Mabunda, Inacio Mandomando, John Aponte, Esperança Sevene, Pedro L Alonso, Clara Menéndez

**Affiliations:** 1 Barcelona Centre for International Health Research, Hospital Clinic, Institut d'Investigacions Biomedicas August Pi i Sunyer (IDIBAPS), Universitat de Barcelona, Barcelona, Spain; 2 The Manhiça Health Research Center (CISM), Manhiça, Mozambique; 3 National Institute of Health, Ministry of Health, Maputo, Mozambique; 4 The National Malaria Control Programme, Ministry of Health, Maputo, Mozambique; 5 School of Medicine, Eduardo Mondlane University, Maputo, Mozambique

## Abstract

**Background:**

There is a widespread notion, based on limited information, that in areas of stable malaria transmission most pregnant women with *Plasmodium falciparum *infection are asymptomatic. This study aim to characterize the clinical presentation of malaria in African pregnant women and to evaluate the adequacy of case management based on clinical complaints.

**Methods:**

A hospital-based descriptive study between August 2003 and November 2005 was conducted at the maternity clinic of a rural hospital in Mozambique. All women attending the maternity clinic were invited to participate. A total of 2,330 women made 3,437 eligible visits, 3129 were analysed, the remainder were excluded because diagnostic results were unavailable or they were repeat visits. Women gave a standardized clinical history and had a medical exam. Malaria parasitaemia and haematocrit in capillary blood was determined for all women with signs or symptoms compatible with malaria including: presence and history of fever, arthromyalgias, headache, history of convulsions and pallor. Outcome measure was association of malaria symptoms or signs with positive blood slide for malaria parasitaemia.

**Results:**

In 77.4% of visits pregnant women had symptoms suggestive of malaria; 23% (708/3129) were in the first trimester. Malaria parasitaemia was confirmed in 26.9% (842/3129) of visits. Headache, arthromyalgias and history of fever were the most common symptoms (86.5%, 74.8% and 65.4%) presented, but their positive predictive values for malaria parasitaemia were low [28% (27–30), 29% (28–31), and 33% (31–35), respectively].

**Conclusion:**

Symptoms suggestive of malaria were very frequent among pregnant women attending a rural maternity clinic in an area of stable malaria transmission. However, less than a third of them were parasitaemic. In the absence of microscopy or rapid diagnostic tests, a large proportion of women, including those in the first trimester of gestation, would be unnecessarily receiving antimalarial drugs, often those with unknown safety profiles for pregnancy. Accessibility to malaria diagnostic tools needs to be improved for pregnant women and drugs with a safety profile in all gestational ages are urgently needed.

## Background

Each year 25 million African women become pregnant in malaria endemic areas [1]. In most of these settings malaria transmission is stable and *Plasmodium falciparum is *predominant [2]. Primigravidae women have the highest risk for malaria infection [3,4]. Malaria infections are associated with maternal anaemia, low birth weight and premature delivery [5-8]. Despite this well-documented indirect morbidity burden, it is generally assumed that due to the acquisition of significant levels of anti-malarial immunity in areas of stable transmission, parasitaemic pregnant women are rarely symptomatic, and that severe disease or death from malaria is extremely unusual [9]. However, this assumption is based on a few studies carried out among women attending antenatal clinics (ANCs) for routine examination and on cross-sectional community studies, which tend to underestimate the actual frequency of malaria-related symptoms [10,11]. This misrepresentation of the problem's magnitude has affected resource prioritisation for malaria control in pregnancy in many African countries http://rbm.who.int/amd2003/amr2003/pdf/ch4.pdf.

Another widely accepted assumption is that women rarely present with malaria-related clinical complaints during the first trimester of pregnancy. As chloroquine (CQ) is being phased out to treat uncomplicated malaria, little attention has been paid to ensuring the availability of antimalarial drugs that could be safely administered during this critical period of pregnancy. The current recommendation for the treatment of malaria episodes in the first trimester is oral quinine for seven days [12]. However, the effectiveness of this regimen is likely to be low due to the frequency of side effects related to quinine and the low compliance with such a lengthy regimen.

A hospital-based descriptive study was carried out in rural Mozambique to document the frequency that pregnant women present with clinical complaints suggestive of malaria and to assess their parasitological confirmation. The study aimed to characterize the clinical presentation of malaria in African pregnant women and to understand the extent that current case management based on clinical complaints is an adequate strategy in pregnancy, especially in the context of new, less safe and more expensive antimalarial drugs.

## Methods

### Study area and population

The study was conducted in Manhiça (Manhiça District, Maputo Province), a rural area in southern Mozambique. The characteristics of the area have been described in detail elsewhere [13]. The area has a population of approximately 82,000 inhabitants, who are under continuous Demographic Surveillance (DSS) carried out by the Centro de Investigação em Saúde de Manhiça (CISM) (Manhica Health Research Center). The adjacent Manhiça Health Centre (MHC) is a 110-bed health facility that offers preventive and curative services. Eighty percent of the pregnant women in this area have an institutional delivery and more than 90% attend the ANC at least once during pregnancy [Nhacolo, personal communication]. The ANC also provides curative services to pregnant women when they are sick.

Perennial malaria transmission with pronounced seasonality is mostly attributable to *P. falciparum. Anopheles funestus *is the main vector, and the estimated entomological inoculation rate for 2002 was 38 infective bites per person per year [14]. In Manhiça, RIII resistance to CQ in children was 30% in 1999 [15]. Previous data from this area showed that sulphadoxine-pyrimethamine (SP) in children had a therapeutic efficacy (adequate clinical response) of 83%, with an in vivo parasitological sensitivity of 83,6% at day 14 [16]. According to data from 2006, the fertility rate is 4.6, the maternal mortality ratio 820 per 100,000 live births and the life expectancy 42.9 (Nhacolo, unpublished). Insecticide-treated net (ITN) coverage at the time of the study was zero [17].

The current Mozambican policy for malaria prevention in pregnancy relies on ITN use and, more recently, intermittent preventive treatment (IPTp) with SP. However, at the time of the study, none of these measures were recommended yet. Malaria control during pregnancy relied solely on presumptive treatment of clinical episodes with CQ or SP in the first and subsequent trimesters for uncomplicated malaria, respectively, and with parenteral quinine for severe malaria.

The study protocol was approved by the National Mozambican Ethics Committee and the Hospital Clinic of Barcelona Ethics Review Committee.

### Study design

Between August 2003 and November 2005, a morbidity surveillance system was established at the MHC maternity clinic, as a passive case detection system, for all women (pregnant, puerperal and women with gynaecological complaints) attending this clinic with clinical complaints (i.e., not for those attending the routine antenatal clinic). During the visit, after verbal informed consent was obtained, the maternity staff recorded the clinical and demographic information onto a standardized questionnaire. Health staff recorded information on age, parity, gestational age, axillary temperature, and signs and symptoms focused on malaria. A capillary blood sample for quantifying *P. falciparum *parasitaemia and haematocrit was collected if women reported at least one pre-defined clinical criteria suggestive of malaria. The clinical criteria included: axillary temperature ≥ 37.5°C, reported history of fever in the last 24 hours, pallor, arthromyalgias, headache and/or history of convulsions. Project staff was available 24 hours a day to identify women and to ensure adequate documentation and clinical management. Women with a blood slide positive for malaria were treated with SP plus CQ in cases of non-complicated malaria (in the first trimester CQ alone was given). Oral quinine for 7 days was given as the second line treatment. Women with complicated malaria were admitted to the maternity ward and treated with parenteral quinine followed by SP. Anaemia was treated following national guidelines with oral ferrous sulphate and folic acid for one month.

### Laboratory methods

Duplicate thick and thin blood films were Giemsa-stained and read to determine parasite species and density of *P. falciparum *asexual stages according to standard, quality-controlled procedures [18]. The haematocrit was measured using a microhaematocrit centrifuge and read in a Hawksley (Lancing, UK) haematocrit reader.

### Statistical methods and definitions

Double data entry, validation and cleaning were done using Microsoft Visual FoxPro 5.0, and statistical analysis was performed using STATA.8.2 (STATA Corporation, College Station, TX, USA). Differences between proportions were compared using the X^2 ^or Fisher's exact test. To evaluate the association between the presence or history of fever, headache, arthromyalgias, convulsions, pallor, age, parity, gestational age, number of previous malaria episodes and season with parasitaemia, multivariate logistic regression models were used. Covariates were selected using a stepwise methodology with a p value of 0.10 for removal and 0.05 for addition to the model. The stratum specific significance tests were yielded by Wald test. Missing values were coded as such and excluded from analysis.

A malaria episode was defined as a *P. falciparum *parasitaemia of any density and signs and/or symptoms suggestive of malaria (fever or history of fever in the last 24 hours, headache, arthromyalgias, and/or pallor), but without severe anaemia, history of convulsions or high-density parasitaemia. For the purpose of the analysis, the duration of any single malaria episode was estimated as 28 days, in order to distinguish between visits on the same episode and a new one. Moderate anaemia in the pregnant woman was defined as a PCV (packed cell volume) between 21% and 32%. Severe anaemia was defined as a PCV between 16% and 20%, and very severe anaemia as a PCV lower than 16%.

**Figure 1:**
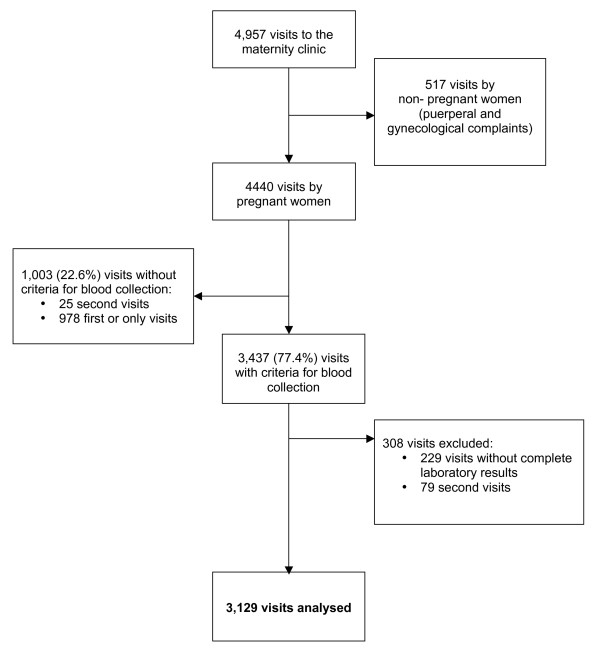
Profile of the study.

During the study period 4957 visits were made to the maternity clinic (Figure 1). Of them 517 (10.4%) were made by non-pregnant women (puerperal and women with gynaecological complaints) and were not included in this analysis. Of the 4440 visits made by pregnant women 3437 (77.4%) visits had criteria for blood collection. Of these, in 229 visits whether the parasitaemia or the haematocrit result was not available and 79 was the second visit of the same episode. This paper reports findings on 3129 first or only visits of the same episode made by pregnant women with criteria for blood collection and complete laboratory results.

## Results

### Characteristics of visits made by pregnant women

Demographic characteristics of visits to the maternity clinic are shown in Table 1. Nearly a quarter of the visits were made in the first trimester, and 12.9% were women in their first pregnancy. More women came during the wet season than in the dry season. Fever or history of fever was found in more than half (65.3%; 2044/3129) of all cases, while current fever was present in only 12.1% (377/3129). Arthromyalgias and headache were reported in 74.8% (2339/3129) and 86.5% (2706/3129) of the visits, respectively. Convulsions were reported in just 0.4% (13/3129) and pallor was found in 7% (218/3129) of the visits (Table 1).

**Table 1:** Characteristics of the pregnant women attending the maternity clinic

N = 3129 *			
		**n**	**%**

**Age category **(years)	**<20**	837	27.0
	**20–34**	1982	63.9
	**≥35**	283	9.1
**Trimester†**	**1st**	708	22.8
	**2nd**	797	25.6
	**3rd**	1605	51.6
**Parity**	**nulliparous**	403	12.9
	**1 to 3**	1896	60.7
	**≥4**	824	26.4
**Season‡**	**Dry season**	1371	43.8
	**Wet season**	1757	56.2

**Signs and symptoms**		**n**	**%**

**Current Fever¶**		377	12.1
Fever or history of fever **		2044	65.4
**Arthromyalgias**		2339	74.8
**Headache**		2706	86.5
**Convulsions**		13	0.4
**Pallor**		218	7.0

* Number of first or only visits made by pregnant women with criteria for blood collection and complete laboratory results

† 1st trimester: 0–12 weeks; 2nd trimester: 13–24 weeks; 3rd trimester: 25–40 weeks

‡ Wet season: November to April; Dry season: May to October

¶Axillary temperature ≥ 37.5°C

** Axillary temperature ≥ 37.5°C and/or history of fever in the last 24 hours

Table 2 shows the proportion of *P. falciparum *parasitaemia and anaemia in the 3,129 visits analysed. Malaria parasites were found in 26.9% (842/3,129) of the visits while overall anaemia was observed in 56.1% (1755/3,129). Severe or very severe anaemia were found in 3.1% (96/3,129) of the visits.

**Table 2:** Proportion of visits with *P falciparum *asexual parasitaemia and anemia

N = 3129 *		
	**n**	**%**

***P falciparum *asexual parasitaemia**	842	26.9
**Moderate anemia†**	1659	53.0
**Severe anemia‡**	77	2.5
**Very severe anemia§**	19	0.6

* Number of first or only visits made by pregnant women with criteria for blood collection and complete laboratory results

† Moderate anemia: hematocrit = 21–32%

‡ Severe anemia: hematocrit = 16–20%

§Very severe anemia: hematocrit ≤15%

### Frequency of visits and malaria episodes per woman

The frequency of visits to the maternity clinic is shown in Table 3. The average number of visits per woman was 1.34 (SD 0.73). Nearly a quarter (23.8%; 554/2,330) of the women came more than once to the clinic. There were 0.36 (SD 0.55) malaria episodes per woman, and over a third (32.9%; 765/2,330) of them had one or more malaria episodes, contributing to a total of 842 episodes. The mean number of visits per malaria episode was 1.09 (SD 0.30). In 75 (8.9%) out of 842 episodes, the women came at least twice. Women were admitted to hospital in 19.1% (597/3,129) of the outpatients visits made to the maternity clinic. In 22 visits (0.7%; 22/3,129) the women were referred to another hospital, and the rest were sent home. Forty-three percent (361/842) of the malaria episodes required hospital admission. Chloroquine was given in 321 (38.1%) of the 842 malaria episodes, quinine in 365 (43.3%), SP in 610 (72.4%) and amodiaquine in 12 (1.4%). Frequently more than one antimalarial drug was prescribed.

**Table 3:** Frequency of visits to the maternity clinic and malaria episodes per woman

N = 2330*			
		**n**	**%**

**Visits per woman**	1 visit	1776	76.2
	2 visits	395	17.0
	≥ 3 visits	159	6.8
**Malaria episodes per woman†**	None	1565	67.2
	1 episode	694	29.8
	2 episodes	65	2.8
	3 episodes	6	0.3
**Visits per malaria episode‡**	1 visit	767	91.1
	2 visits	72	8.6
	3 visits	3	0.4

*Number of pregnant women with criteria for blood collection and complete laboratory results

† Defined as *P falciparum *parasitaemia plus criteria for blood collection. Total of 842 malaria episodes

### Risk factors associated with P. falciparum clinical malaria in pregnant women

In both the univariate and multivariate analyses, younger age, primigravidae, second and third trimester of gestational age and wet season were all statistically significantly associated with an increased risk of clinical malaria (Table 4). Having had a previous malaria episode was also independently associated with a significantly increased risk of malaria parasitaemia. The prevalence of visits with presence of signs and/or symptoms suggestive of malaria increased significantly with increased parasite density.

**Table 4:** *P falciparum *malaria in pregnant women

N = 3129*		**Univariate OR†**	**(95%CI)**	**Multivariate OR‡**	**(95%CI)**
**Age category **(years)	**<20**	1.00	--	1.00	--
	**20–34**	0.58	(0.49–0.70)	0.70	(0.57–0.86)
	**≥35**	0.42	(0.30–0.58)	0.58	(0.38–0.87)
**Parity**	**nulliparous**	1.00	--	1.00	--
	**1 to 3**	0.62	(0.50–0.78)	0.75	(0.58–0.96)
	**4 or >**	0.43	(0.33–0.56)	0.65	(0.47–0.91)
**Season§**	**Dry season**	1.00	--	1.00	--
	**Wet season**	1.67	(1.42–1.97)	1.63	(1.37–1.93)
**Trimester ¶**	**1st**	1.00	--	1.00	--
	**2nd**	1.91	(1.49–2.43)	1.87	(1.45–2.42)
	**3rd**	1.80	(1.44–2.24)	1.82	(1.44–2.28)
**Previous malaria episodes**		1.44	(1.11–1.85)	1.37	(1.05–1.79)

* Number of first or only visits made by pregnant women with criteria for blood collection and complete laboratory results

† OR = Odds Ratio

‡ Global model considering fever or history of fever

§ Wet season: November to April; Dry season: May to October

¶ 1st trimester: 0–12 weeks; 2nd trimester: 13–24 weeks; 3rd trimester: 25–40 weeks

Table 5 shows the prevalence and positive predictive values (the probability that a person with a positive test, in this case presenting a sign or symptoms, is a true positive) of analyzed signs and symptoms in relation to *P. falciparum *parasitaemia. Generally, all signs and symptom had low predictive values (less than 40%) with the exception of current fever (PPV 50% 95% CI 45–56%). However, the prevalence of this sign was low (12%). The association of signs and symptoms with malaria parasitaemia did not vary when the analysis was done by gestational age (data not shown). Combining arthromyalgias, headache and fever or history of fever did not improve the positive predictive value of parasitaemia (PPV 35% 95%CI 33–37%).

**Table 5:** Positive predictive value of clinical signs and symptoms for *P falciparum *parasitaemia in 3129* visits made by pregnant women

**Signs/symptoms**	**n**	**%**	**PPV† (%)**	**(95%CI)**
**Current fever‡**	377	12.1	50	(45–56)
**Fever or history of fever§**	2044	65.4	33	(31–35)
**Arthromyalgias**	2339	74.8	29	(28–31)
**Headache**	2706	86.5	28	(27–30)
**Convulsions**	13	0.4	23	(5.0–54)
**Pallor**	218	7.0	27	(21–33)

* Number of first or only visits of pregnant women with criteria for blood collection and complete laboratory results

† Positive Predictive Value

‡ Axillary temperature ≥ 37.5°C

§Axillary temperature ≥ 37.5°C and/or history of fever in the last 24 hours

## Discussion

In this rural area of Mozambique the majority (77.4%) of pregnant women attending the maternity clinic with clinical complaints had signs or symptoms suggestive of malaria. However, less than a third (26.9%) of cases had microscopically confirmed parasitaemia. Nearly a quarter of the clinic visits with an illness indicative of malaria were made by women in their first trimester of pregnancy. These findings are relevant given the limited resources dedicated to healthcare in rural Africa, because the majority of pregnant women do not have access to parasitological diagnosis, and they usually receive presumptive treatment, leading to unnecessary drug exposure.

Primigravidae women made fewer visits to the maternity clinic than other parities. This may be due to sociological factors such as awareness of the risks of being sick during pregnancy or lower social capacity for decision-making. There were more women attending the clinic during the rainy season, suggesting that malaria has a role in maternal morbidity in this area.

One out of four women visited the clinic more than once during the same pregnancy, indicating that morbidity may be concentrated in a specific group of individuals. This has been observed in children in relation to malaria [18]. Nearly 10% of women came more than once within the same confirmed malaria episode. This may be due to recrudescence of the initial parasitaemia, although this can not be confirmed without molecular analysis. Consistent with previous reports, these findings showed that younger age, lower parity and wet season were independently associated with an increased risk of *P. falciparum *parasitaemia, while first trimester was associated with a reduced risk [11,19-22].

Information about the clinical presentation of malaria episodes during pregnancy in stable transmission areas is surprisingly limited or incomplete [10,23]. Most of the data comes from low endemic areas [24-26]. Furthermore, there is a lack of information on the association between peripheral parasitaemia and the presence of signs and symptoms of malaria during pregnancy. A study carried out in a stable transmission area of Sudan suggested that maternal history can accurately predict malaria episodes during pregnancy, justifying the presumptive treatment of the clinical presentation [27].

In contrast, these findings suggest the opposite. All clinical signs and symptoms evaluated had low positive predictive values. Only the presence of fever had a PPV of 50% (95% CI 45–56%), however its prevalence among women was very low. The majority of women presenting a sign or symptom suggestive of malaria did not have parasitaemia, and clinical complaints had a relatively low value in guiding the treatment of malaria in the absence of parasitological diagnosis. The simultaneous occurrence of the most frequently presented signs and symptoms (headache, arthromyalgias and history of recent fever) did not substantially increase the PPV (35%, 95% CI 33–37%) for malaria parasitaemia. This study was focused in women who presented with predefined symptoms, and thus it was not possible to calculate their sensitivity and specificity. This may be seen as a limitation of the study. However, the aim was to describe the clinical presentation of malaria parasitaemia in pregnant women attending the maternity clinic because they have clinical complaints. Asymptomatic pregnant women although may also be parasitaemic, are unlikely to attend a health facility except for antenatal routine control visits.

## Conclusion

Although presumptive malaria treatment has been recommended by WHO as a malaria control measure in endemic areas [12,28], this strategy should be reconsidered for malaria in pregnancy given the lack of available antimalarial drugs with acceptable safety profiles. This is especially critical during the first trimester due to concerns from animal data about the safety of artemisinin derivatives in pregnancy [29]. The current recommendation is to give artemisinin-based combinations (ACTs) as the first line treatment for uncomplicated malaria for women in the second and third trimesters and as second line treatment in the first trimester [12]. Careful evaluation of drug safety is urgent as countries adopt this policy.

Due to the absence of safe alternatives to failing drugs, case management of uncomplicated malaria during pregnancy poses one of the greatest challenges to malaria control. Increasing the accessibility to diagnostic tools is needed to identify positive cases and to avoid unnecessary administration of drugs. The use of rapid diagnostic tests (RDTs) is one option to reduce overtreatment in areas where microscopy facilities are absent. RDTs are being increasingly promoted, however, their implementation is still limited by their cost, and they will have distribution and life span issues that need consideration. As we improve the tools for malaria control, finding antimalarials that are safe for pregnant women with no restriction by gestational age remains a public health priority.

## Authors' contributions

The study was conceived by CM and this paper drafted by CM and AB. It was conducted by AB and CM with substantial contributions from CR, BS, IM and ES. Data analyses were conducted by LB, and supervised by SS and JA. PA and SM participated in the overall running of the study, and contributed to the interpretation of data and by giving critical revision of the final draft. All authors read and approved the final version.

## References

[B1] WHO/AFRO (2004). A strategic framework for malaria prevention and control during pregnancy in the African region.

[B2] Steketee RW, Nahlen BL, Parise ME, Menendez C (2001). The burden of malaria in pregnancy in malaria-endemic areas. Am J Trop Med Hyg.

[B3] Steketee RW, Wirima JJ, Slutsker L, Heymann DL, Breman JG (1996). The problem of malaria and malaria control in pregnancy in sub-Saharan Africa. Am J Trop Med Hyg.

[B4] Menendez C (2006). Malaria during pregnancy. Curr Mol Med.

[B5] Greenwood BM, Greenwood AM, Snow RW, Byass P, Bennett S, Hatibnjie AB (1989). The Effects of Malaria Chemoprophylaxis Given by Traditional Birth Attendants on the Course and Outcome of Pregnancy. Trans R Soc Trop Med Hyg.

[B6] DAlessandro U, Langerock P, Bennett S, Frances N, Cham K, Greenwood BM (1996). The impact of a national impregnated bed net programme on the outcome of pregnancy in primigravidae in The Gambia. Trans R Soc Trop Med Hyg.

[B7] Menendez C, Ordi J, Ismail MR, Ventura PJ, Aponte JJ, Kahigwa E, Font F, Alonso PL (2000). The impact of placental malaria on gestational age and birth weight. J Infect Dis.

[B8] Brabin B (1991). An Assessment of Low-Birth-Weight Risk in Primiparae As An Indicator of Malaria Control in Pregnancy. Int J Epidemiol.

[B9] Nosten F, Rogerson SJ, Beeson JG, McGready R, Mutabingwa TK, Brabin B (2004). Malaria in pregnancy and the endemicity spectrum: what can we learn?. Trends Parasitol.

[B10] Steketee RW, Wirima JJ, Slutsker L, Khoromana CO, Heymann DL, Breman JG (1996). Malaria treatment and prevention in pregnancy: Indications for use and adverse events associated with use of chloroquine or mefloquine. Am J Trop Med Hyg.

[B11] Saute F, Menendez C, Mayor A, Aponte J, Gomez-Olive X, Dgedge M, Alonso PL (2002). Malaria in pregnancy in rural Mozambique: the role of parity, submicroscopic and multiple Plasmodium falciparum infections. Trop Med Int Health.

[B12] World Health Organization (WHO) (2006). Guidelines for the treatment of malaria.

[B13] INDEPTH Network (2006). Population and health in developing countries.

[B14] Alonso PL, Sacarlal J, Aponte JJ, Leach A, Macete E, Milman J, MandoMando I, Bassat Q, Guinovart C, Espasa M, Corachan S, Lievens M, Navia MM, Dubois MC, Menéndez C, Dubovsky F, Cohen J, Thompson R, Ballou WR (2004). Efficacy of the RTS,S/AS02A vaccine against Plasmodium falciparum infection and disease in young African children: randomised controlled trial. Lancet.

[B15] Mayor AG, Gomez-Olive X, Aponte JJ, Casimiro S, Mabunda S, Dgedge M, Barreto A, Alonso PL (2001). Prevalence of the K76T mutation in the putative Plasmodium falciparum chloroquine resistance transporter (pfcrt) gene and its relation to chloroquine resistance in Mozambique. J Infect Dis.

[B16] Abacassamo F, Enosse S, Aponte JJ, Gomez-Olive FX, Quinto L, Mabunda S, Barreto A, Magnussen P, Ronn AM, Thompson R, Alonso PL (2004). Efficacy of chloroquine, amodiaquine, sulphadoxine-pyrimethamine and combination therapy with artesunate in Mozambican children with non-complicated malaria. Trop Med Int Health.

[B17] Macete E, Aide P, Aponte JJ, Sanz S, Mandomando I, Espasa M, Sigauque B, Dobaño C, Mabunda S, Dge Dge M, Alonso PL, Menéndez C (2006). Intermittent preventive treatment for malaria control administered at the time of routine vaccinations in Mozambican infants: a randomized, placebo-controlled trial. J Infect Dis.

[B18] Alonso PL, Smith T, Schellenberg JRMA, Masanja H, Mwankusye S, Urassa H, Bastos de Azebedo I, Chongela J, Kobero S, Menéndez C (1994). Randomized Trial of Efficacy of Spf66 Vaccine Against Plasmodium-Falciparum Malaria in Children in Southern Tanzania. Lancet.

[B19] Rogerson SJ, Van den Broek NR, Chaluluka E, Qongwane C, Mhango CG, Molyneux ME (2000). Malaria and anaemia in antenatal women in Blantyre, Malawi a twelve-month survey. Am J Trop Med Hyg.

[B20] Leenstra T, Phillips-Howard PA, Kariuki SK, Hawley WA, Alaii JA, Rosen DH, Oloo AJ, Nahlen BL, Kager PA, ter Kuile FO (2003). Permethrin-treated bed nets in the prevention of malaria and anaemia in adolescent schoolgirls in western Kenya. Am J Trop Med Hyg.

[B21] Dicko A, Mantel C, Thera MA, Doumbia S, Diallo M, Diakite M, Sagara I, Doumbo OK (2003). Risk factors for malaria infection and anaemia for pregnant women in the Sahel area of Bandiagara, Mali. Acta Trop.

[B22] Tako E, Zhou A, Lohoue J, Leke R, Taylor D, Leke R (2005). Risk factors for placental malaria and its effect on pregnancy outcome in Yaounde, Cameroon. Am J Trop Med Hyg.

[B23] Diagne N, Rogier C, Cisse B, Trape JF (1997). Incidence of clinical malaria in pregnant women exposed to intense perennial transmission. Trans R Soc Trop Med Hyg.

[B24] Nosten F, TerKuile F, Maelankirri L, Decludt B, White NJ (1991). Malaria During Pregnancy in An Area of Unstable Endemicity. Trans R Soc Trop Med Hyg.

[B25] Looareesuwan S, White NJ, Karbwang J, Turner RC, Phillips RE, Kietinun S, Karbwaang J, Rackow C, Turner RC, Warrell DA (1985). Quinine and Severe Falciparum-Malaria in Late Pregnancy. Lancet.

[B26] Singh N, Shukla MM, Sharma VP (1999). Epidemiology of malaria in pregnancy in central India. Bulletin of the World Health Organization.

[B27] Taha T (1996). Comparison of reported and confirmed malaria during pregnancy: findings from hospital and community studies in Sudan. Afr Med J.

[B28] World Health Organization (1986). WHO expert committee on malaria 18th report. World Health Organ Tech Rep Ser.

[B29] Clark RL, White TE, Clode A, Gaunt I, Winstanley P, Ward SA (2004). Developmental toxicity of artesunate and an artesunate combination in the rat and rabbit. Birth Defects Res B Dev Dev Reprod Toxicol.

